# Prognostic factors of pediatric pelvic and genitourinary rhabdomyosarcoma: An analysis based on SEER database

**DOI:** 10.3389/fonc.2022.992738

**Published:** 2022-09-05

**Authors:** Jiheng Wu, Xinyi Shou, Jiabin Cai, Junqing Mao, Jianqin Qian, Jinhu Wang, Shaoqing Ni

**Affiliations:** ^1^ National Clinical Trial Institute, The Children’s Hospital, Zhejiang University School of Medicine, National Clinical Research Center for Child Health, Hangzhou, China; ^2^ The Children’s Hospital, Zhejiang University School of Medicine, National Clinical Research Center for Child Health, Hangzhou, China; ^3^ Department of Surgical Oncology, The Children’s Hospital, Zhejiang University School of Medicine, National Clinical Research Center for Child Health, Hangzhou, China; ^4^ Research Center for Clinical Pharmacy, Zhejiang University, Hangzhou, China

**Keywords:** pediatric rhabdomyosarcoma, prognostic survival analysis, retrospective study, radiation therapy, outcomes assessment

## Abstract

**Background:**

Rhabdomyosarcoma (RMS) is the most common soft-tissue sarcomas in children. This study aimed to investigate the prognostic factors of pelvic and genitourinary RMS in children and evaluate the survival outcomes of these children treated with or without radiation therapy (RT).

**Methods:**

The Surveillance, Epidemiology, and End Results Program (SEER) database was required for children with pelvic and genitourinary RMS. Overall survival (OS) and cancer-specific survival (CSS) were analyzed using the Kaplan-Meier method, log-rank test, Cox proportional hazards models, and propensity score-matched analyses.

**Results:**

For the 262 patients analyzed, the most common biological subtypes were embryonic (n=209, 79.8%) and alveolar (n=29, 11.1%). Patients with alveolar RMS had the worst prognosis (P < 0.05). The testis (n=122, 46.6%) was the most common location, followed by the urinary bladder (n=57, 21.8%) and prostate (n=48, 18.3%). Uterus RMS had the highest survival rate, followed by testis, urinary bladder, and prostate RMS. Favorable prognostic factors were age at diagnosis < 15 years, non-alveolar histological subtype, early tumor stage (localized/regional), specific sites (uterus and testis), and treatment (cancer-directed surgery and chemotherapy) (P < 0.05). Propensity score-matched analyses comparing the cohorts of patients treated with or without RT demonstrated no significant differences in prognostic survival (OS: P=0.872, CSS: P=0.713).

**Conclusion:**

The nomogram constructed based on independent prognostic factors may accurately predict survival rates at 1 and 5 years. Surgery and adjuvant chemotherapy can be effective treatments, but RT fails to guarantee a survival benefit. Therefore, prospective trials evaluating RT for pediatric pelvic and genitourinary RMS are warranted.

## Introduction

Rhabdomyosarcoma (RMS) is the most common soft tissue sarcoma in children and adolescents aged 0-19 years (1). Pelvic and genitourinary RMS accounts for approximately 27% of all pediatric RMS (2). The pelvis and genitourinary organs are close to the digestive, reproductive, and urinary organs. These adjacent organs might be affected during RMS treatments (surgery, iliac artery chemotherapy, and radiation therapy (RT)), which may result in unsatisfactory treatment outcomes (3–6). Although existing guidelines recommend RT for patients with RMS, there are no available studies mentioning the prognosis of children with pelvic and genitourinary RMS receiving RT. This study selected pediatric patients with pelvic and genitourinary RMS from the Surveillance, Epidemiology, and End Results Program database (SEER, 1975-2016). Clinical features (sex, age, race, tumor site, and pathological type) and treatment methods (surgery, iliac artery chemotherapy, and RT) were used to determine the prognostic factors and assess prognostic survival.

## Methods

The SEER database of the National Cancer Institute covers 26% of the incidence and survival data from 17 population-based cancer registries in the United States (7). We identified and included all patients aged 0-19 from the SEER database 1975-2016 who were histologically diagnosed with RMS (International Classification of Disease for Oncology [ICD-O-3] code ‘8900/3: Rhabdomyosarcoma, NOS’, ‘8901/3: Pleomorphic rhabdomyosarcoma,’ ‘8902/3: mixed type rhabdomyosarcoma,’ ‘8910/3: Embryonal rhabdomyosarcoma,’ ‘8912/3: Spindle cell rhabdomyosarcoma,’ ‘8920/3: Alveolar rhabdomyosarcoma,’ ‘8921/3: rhabdomyosarcoma with ganglionic differentiation’). Primary site-specific codes included RMS originating in the retroperitoneal pelvic area. Patient data extracted from the SEER database included demographic, pathological, and clinical variables. The demographic variables included sex and age at diagnosis. Pathologic variables included tumor histologic subtype, primary site, and extent of disease, as evaluated using collaborative stage coding methods. Clinical variables included chemotherapy (yes/no), RT (yes/no), surgery (yes/no), overall survival (OS), and cancer-specific survival (CSS).

This study was exempt from local research ethics committee approval, considering that SEER data were de-identified and publicly available for research use.

### Statistical analysis

OS was defined as the time from diagnosis to death due to any cause. CSS was defined as the time from diagnosis to death due to pelvic and genitourinary RMS. Median survival time was defined as the length of time when half of the patients died. Kaplan-Meier univariate analysis was performed to calculate the OS and CSS curves (8). Least absolute shrinkage and selection operator (LASSO) regression was performed to select the initial factors and prevent overfitting of the multifactorial models. Covariates were assessed using multivariable Cox proportional hazard regression models with corresponding 95% confidence intervals (CIs).

The performance and discriminative power of prognostic factors were assessed using the concordance index (C-index) values and receiver operating characteristic curve (AUC), which was then visualized as nomograms using the R package “rms” (9). Propensity score analysis was performed to minimize selection bias because of the retrospective nature of the data analysis (10). The 110 propensity score-matched cases were evaluated using univariate and multivariate Cox regression analyses to identify the factors associated with treatment outcomes (11). Covariates were considered statistically significant at P <0.05. Statistical analyses were performed using the R statistical software (version 4.1.1).

## Results

### Demographic and clinical characteristics of study patients

**Figure 1:**
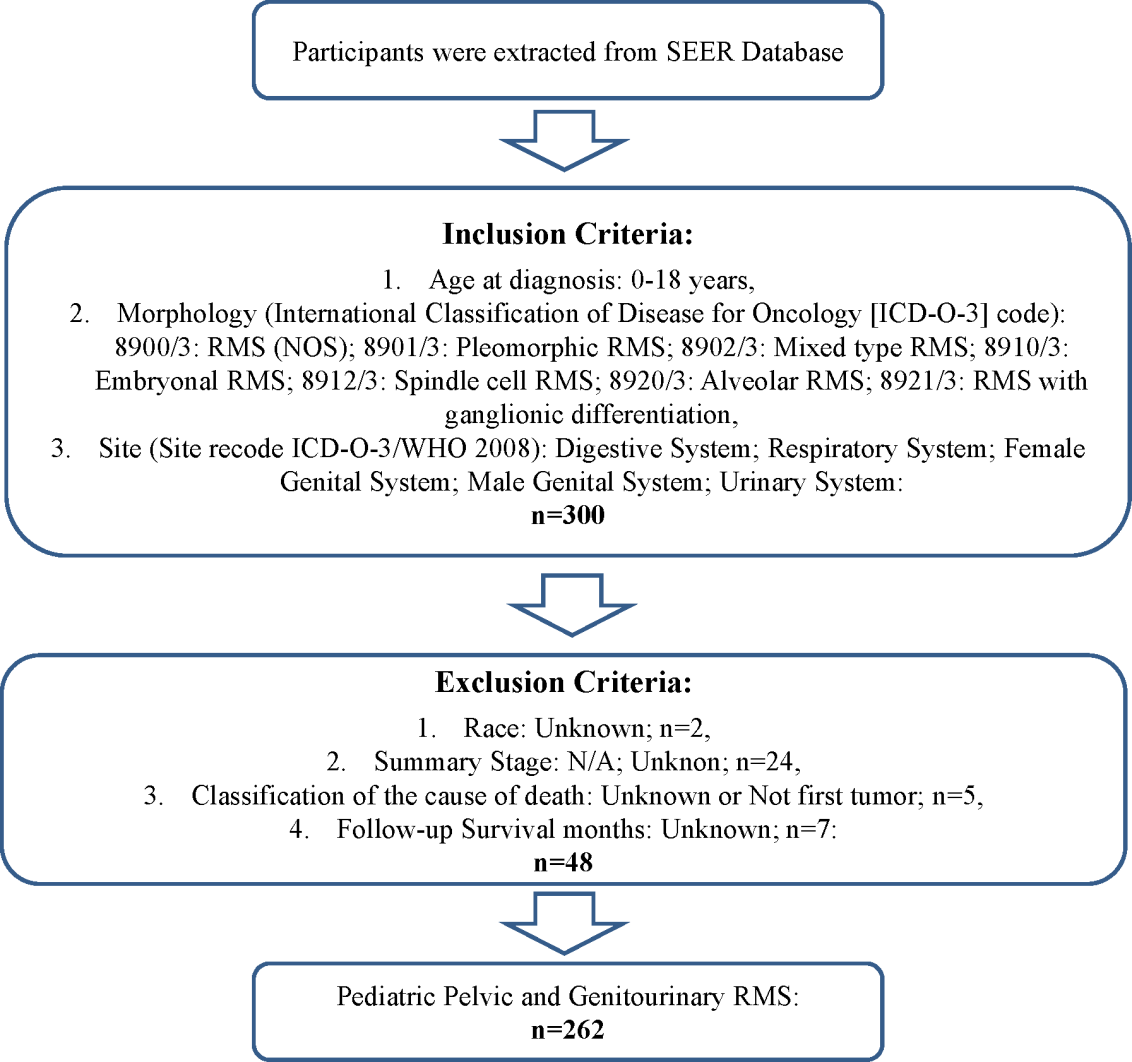
Flowchart of inclusion and exclusion criteria for patient selection.

A total of 262 patients pathologically diagnosed with pelvic and genitourinary RMS were obtained from the SEER database. The eligibility criteria and demographic characteristics are shown in **Figure 1** and **Table 1**. Pelvic and genitourinary RMS were more common in men (79.4%) than in women (20.6%). The testis was the most common primary site (46.6%, n=122), followed by the urinary bladder (21.8%, n=57) and prostate (18.3%, n = 48), and the uterus and other sites made up the remaining 13.3%. Embryons were the most common histological subtype (79.7%, 209), followed by alveolar (11.1%, 29), spindle, and other (9.2%) subtypes. Most patients (96.6%) received chemotherapy, 77.5% were treated with cancer-directed surgery, and nearly half of the patients received RT. The tumor stage was categorized as localized, regional, or distant. Localized tumors (43.9%, 115) included single or multifocal invasive tumors confined to the primary site or in but not beyond the capsule. Regions (30.9%, 81) included direct extension into peripheral tissues, such as blood vessels. Distant (25.2%, 66) included metastasis and invasion of distant lymph nodes, bones, etc.

**Table 1:** Patient characteristics.

	Alveolar RMS (N = 29)	Embryona lRMS (N = 209)	ganglionic differentiation RMS (N = 2)	Mixed RMS (N = 8)	Pleomorphic RMS (N = 2)	Spindle RMS (N = 12)	Overall(N = 262)
**Sex**							
Female	8 (27.6%)	43 (20.6%)	1 (50.0%)	0 (0%)	1 (50%)	1 (8.3%)	54 (20.6%)
Male	21 (72.4%)	166 (79.4%)	1 (50.0%)	8 (100%)	1 (50.0%)	11 (91.7%)	208 (79.4%)
**Age**							
0-4	8 (27.6%)	87 (41.6%)	1 (50.0%)	4 (50.0%)	0 (0%)	6 (50.0%)	106 (40.5%)
5-9	3 (10.3%)	39 (18.7%)	1 (50.0%)	1 (12.5%)	0 (0%)	2 (16.7%)	46 (17.6%)
10-14	5 (17.2%)	35 (16.7%)	0 (0%)	1 (12.5%)	0 (0%)	1 (8.3%)	42 (16.0%)
15-19	13 (44.8%)	48 (23.0%)	0 (0%)	2 (25.0%)	2 (100%)	3 (25.0%)	68 (26.0%)
**Race**							
Black	8 (27.6%)	40 (19.1%)	0 (0%)	1 (12.5%)	0 (0%)	3 (25.0%)	52 (19.8%)
Other	1 (3.4%)	11 (5.3%)	0 (0%)	0 (0%)	0 (0%)	1 (8.3%)	13 (5.0%)
White	20 (69.0%)	155 (74.2%)	2 (100%)	7 (87.5%)	2 (100%)	8 (66.7%)	194 (74.0%)
Unknown	0 (0%)	3 (1.4%)	0 (0%)	0 (0%)	0 (0%)	0 (0%)	3 (1.1%)
**Site**							
Anus, Anal Canal and Anorectum	1 (3.4%)	0 (0%)	0 (0%)	1 (12.5%)	0 (0%)	0 (0%)	2 (0.8%)
Kidney and Renal Pelvis	1 (3.4%)	1 (0.5%)	0 (0%)	0 (0%)	0 (0%)	0 (0%)	2 (0.8%)
Prostate	11 (37.9%)	37 (17.7%)	0 (0%)	0 (0%)	0 (0%)	0 (0%)	48 (18.3%)
Rectum	1 (3.4%)	0 (0%)	0 (0%)	0 (0%)	0 (0%)	0 (0%)	1 (0.4%)
Testis	8 (27.6%)	94 (45.0%)	1 (50.0%)	7 (87.5%)	1 (50.0%)	11 (91.7%)	122 (46.6%)
Urinary Bladder	2 (6.9%)	55 (26.3%)	0 (0%)	0 (0%)	0 (0%)	0 (0%)	57 (21.8%)
Vulva	5 (17.2%)	1 (0.5%)	0 (0%)	0 (0%)	0 (0%)	1 (8.3%)	7 (2.7%)
Ovary	0 (0%)	2 (1.0%)	1 (50.0%)	0 (0%)	1 (50.0%)	0 (0%)	4 (1.5%)
Uterus	0 (0%)	19 (9.1%)	0 (0%)	0 (0%)	0 (0%)	0 (0%)	19 (7.3%)
**Summary stage**							
Distant	15 (51.7%)	47 (22.5%)	0 (0%)	2 (25.0%)	1 (50.0%)	1 (8.3%)	66 (25.2%)
Localized	5 (17.2%)	100 (47.8%)	1 (50.0%)	2 (25.0%)	0 (0%)	7 (58.3%)	115 (43.9%)
Regional	9 (31.0%)	62 (29.7%)	1 (50.0%)	4 (50.0%)	1 (50.0%)	4 (33.3%)	81 (30.9%)
**Cancer-directed surgery**							
No (1)	11 (37.9%)	41 (19.6%)	1 (50.0%)	0 (0%)	0 (0%)	1 (8.3%)	54 (20.6%)
Recommended but not performed (2)	1 (3.4%)	4 (1.9%)	0 (0%)	0 (0%)	0 (0%)	0 (0%)	5 (1.9%)
Yes	17 (58.6%)	164 (78.5%)	1 (50.0%)	8 (100%)	2 (100%)	11 (91.7%)	203 (77.5%)
**Radiation recode**							
Only after surgery	12 (41.4%)	62 (29.7%)	0 (0%)	4 (50.0%)	2 (100%)	3 (25.0%)	83 (31.7%)
None	8 (27.6%)	106 (50.7%)	2 (100%)	4 (50.0%)	0 (0%)	8 (66.7%)	128 (48.9%)
Radiation (without surgery)	9 (31.0%)	36 (17.2%)	0 (0%)	0 (0%)	0 (0%)	1 (8.3%)	46 (17.6%)
before and After surgery	0 (0%)	1 (0.5%)	0 (0%)	0 (0%)	0 (0%)	0 (0%)	1 (0.4%)
Intraoperative radiation	0 (0%)	1 (0.5%)	0 (0%)	0 (0%)	0 (0%)	0 (0%)	1 (0.4%)
Prior to surgery	0 (0%)	3 (1.4%)	0 (0%)	0 (0%)	0 (0%)	0 (0%)	3 (1.1%)
**Chemotherapy**							
Yes	29 (100%)	200 (95.7%)	2 (100%)	8 (100%)	2 (100%)	12 (100%)	253 (96.6%)
No	0 (0%)	9 (4.3%)	0 (0%)	0 (0%)	0 (0%)	0 (0%)	9 (3.4%)

(1) No (surgery): cancer-directed surgery was not performed because it was not recommended by physician due to patient risk factors;

(2) Recommended but not performed: cancer-directed surgery was recommended by the patient’s physician but was not performed as part of the therapy because it was refused by the patients or patients’ guardian or some other reason.

### Feature selection and prognostic signature building

**Figure 2:**
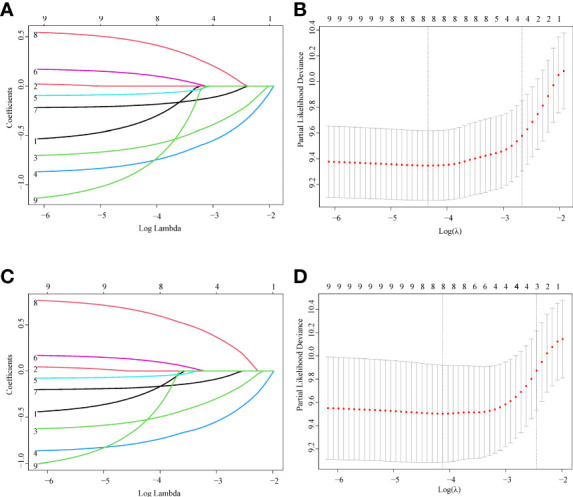
Feature selection using LASSO Cox regression. **(A)** OS: The binomial deviance is plotted versus log (λ). **(B)** OS: LASSO coefficient profiles of the eight clinical features. A coefficient profile plot is produced versus the log (λ). **(C)** CSS: The binomial deviance is plotted versus log (λ). **(D)** CSS: LASSO coefficient profiles of the eight clinical features. A coefficient profile plot is produced versus the log (λ).

In total, nine variables (sex, age, race, site, histology, summary stage, chemotherapy, cancer-directed surgery, and RT) were included in the analysis. According to the LASSO Cox regression analysis results, eight variables (sex, age, site, histology, summary stage, chemotherapy, cancer-directed surgery, and RT) were identified as potential risk factors for OS and CSS (**Figure 2**).

### Identification of independent prognostic factors

A multivariable Cox regression model was used to search for OS- and CSS-related prognostic factors (**Table 2**). According to multivariable Cox analyses, age, site, summary stage, histology, cancer-directed surgery, and chemotherapy were significantly associated with OS (*P* < 0.05), while age, site, summary stage, and cancer-directed surgery were significantly associated with CSS (*P* < 0.05). These variables were defined as the independent prognostic factors for OS and CSS.

**Table 2:** Multivariable Cox regression analysis of predictors of OS and CSS for pelvic and genitourinary RMS.

Risk factor	Overall Survival		Cancer-Specific Survival		
	HR (1) (95% CI)	*P*	HR (1) (95% CI)		*P*
external beam radiotherapy					
Yes	0.947 (0.488 - 1.839)	0.872	1.145 (0.556- 2.358)		0.713
No	Reference				
**Cancer-directed surgery**					
Yes	1.404 (0.643- 2.923)	0.365	1.405 (0.643- 3.069)		0.394
**Recommended but not performed**	3.382 (1.072- 10.670)	**0.038**	3.973 (1.231- 12.825)		**0.021**
No	Reference				
**Chemotherapy**					
** Yes**	0.221 (0.056, 0.878)	**0.032**	0.270 (0.053, 1.380)		0.116
No	Reference				
**Histology Subtype**					
Embryonal RMS	1.312 (0.622- 2.765)	0.475	1.194 (0.547- 2.606)		0.657
**Others (**2)	3.356 (1.016, 11.087)	**0.047**	3.213 (0.949, 10.879)		0.061
Alveolar	Reference				
**Summary Stage**					
** Localized**	0.065 (0.022- 0.192)	**<0.001**	0.070 (0.022- 0.219)		**<0.001**
** Regional**	0.271 (0.140- 0.523)	**<0.001**	0.277 (0.138- 0.555)		**<0.001**
Distant	Reference				
**Site**					
Prostate	1.225 (0.352, 4.270)	0.750	1.107 (0.300, 4.081)		0.878
** Testis**	0.150 (0.039, 0.584)	**0.006**	0.165 (0.040, 0.682)		**0.013**
Urinary Bladder	0.429 (0.132, 1.388)	0.158	0.468 (0.137, 1.599)		0.226
** Uterus**	0.090 (0.435, 0.822)	**0.0329**	–	–
Others (3)	Reference				
Age					
10-14	2.019 (0.793- 5.142)	0.141	1.577 (0.570- 4.362)		0.381
** 15-19**	2.984 (1.442- 6.175)	**0.003**	2.370 (1.257- 5.863)		**0.011**
5-9	1.088 (0.447- 2.724)	0.857	1.130 (0.447- 2.856)		0.795
0-4	Reference				
Gender					
Male	0.577 (0.216, 1.539)	0.356	0.625 (0.231, 1.695)		0.356
Female	Reference				

(1) HR, Hazard ratio.

(2) Others: Ganglionic differentiation RMS; Mixed RMS; Pleomorphic RMS; Spindle RMS.

(3) Others: Anus, Anal Canal and Anorectum; Kidney and Renal Pelvis; Rectum; Vulva; Ovary. Bold values means P value <;0.05.

### Nomogram construction and validation

**Figure 3:**
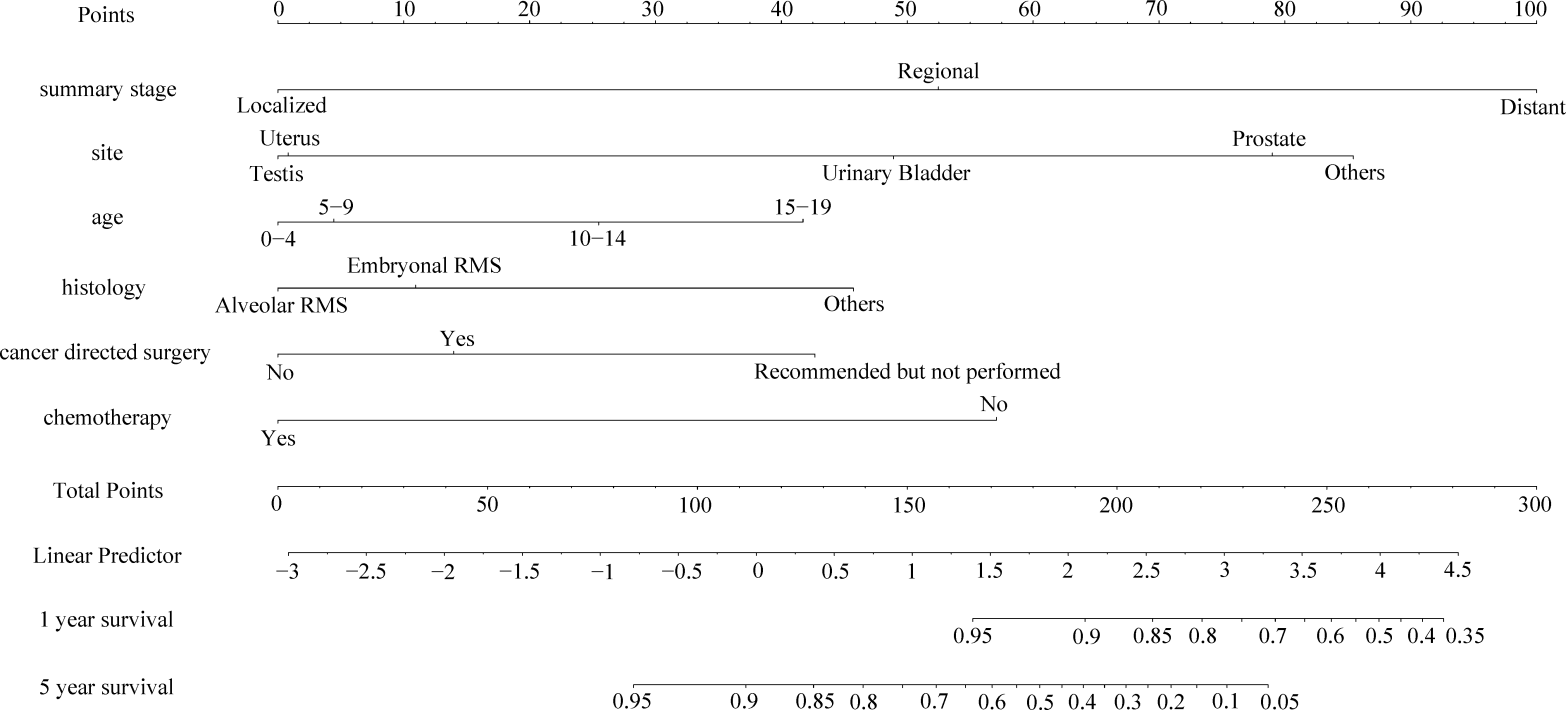
The nomogram of predicting OS of patients aged 0-19 years with pelvic and genitourinary RMS.

**Figure 4:**
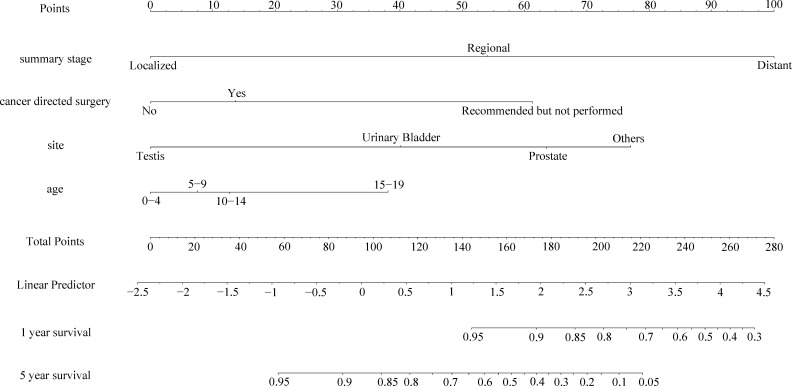
The nomogram of predicting CSS of patients aged 0-19 years with pelvic and genitourinary RMS.

**Figure 5:**
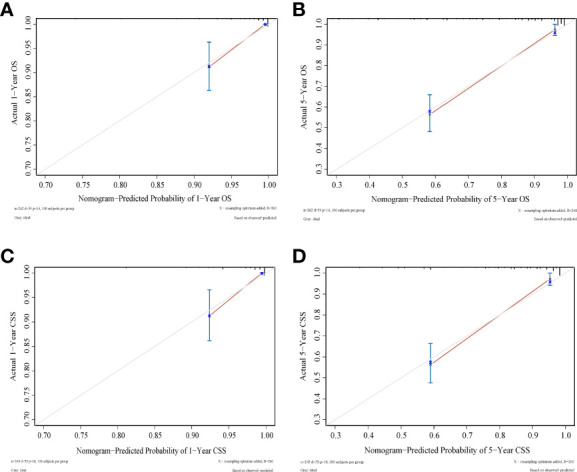
Calibration curves to predict **(A)** 1-year OS; **(B)** 5-year OS; **(C)** 1-year CSS; **(D)** 1-year CSS. Predicted survival is plotted on the x‐axis, and actual survival is plotted on the *y*‐axis.

**Figure 6:**
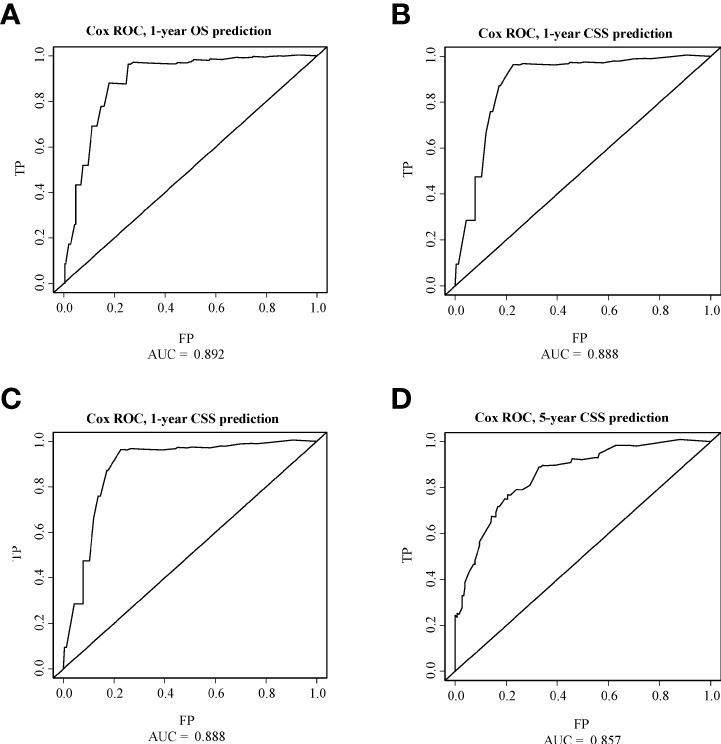
ROC curves of OS and CSS. **(A)** The ROC curve of 1-year OS; **(B)** The ROC curve of 5-year OS; **(C)** The ROC curve of 1-year CSS; **(D)** The ROC curve of 5-year CSS.

As shown in **Figures 3**, **4**, we constructed nomograms by incorporating prognostic factors to predict the 1- and 5-year OS and CSS. The predicted nomogram showed excellent consistency with actual survival outcomes. The accuracy of the nomogram was evaluated using the C-index and AUC values of the ROC. The C-index for OS nomogram was 85.13% (95% CI: 83.06%-87.20%) and for CSS nomogram was 86.45% (95% CI: 82.43%-86.91%). The calibration curve revealed substantial concordance between the actual observation and prediction (**Figure 5**). The AUC values of the 1- and 5-year OS/CSS were 0.892/0.887, and 0.873/0.857, respectively (**Figure 6**). These results indicate that the nomograms showed excellent predictive performance and calibration.

### Survival analysis of different prognostic factors

Survival time data were analyzed for each variable, and the median follow-up was 5.1 years. Kaplan-Meier survival curves were constructed, and the median OS (half of the time of death) was calculated for each variable.

**Figure 7:**
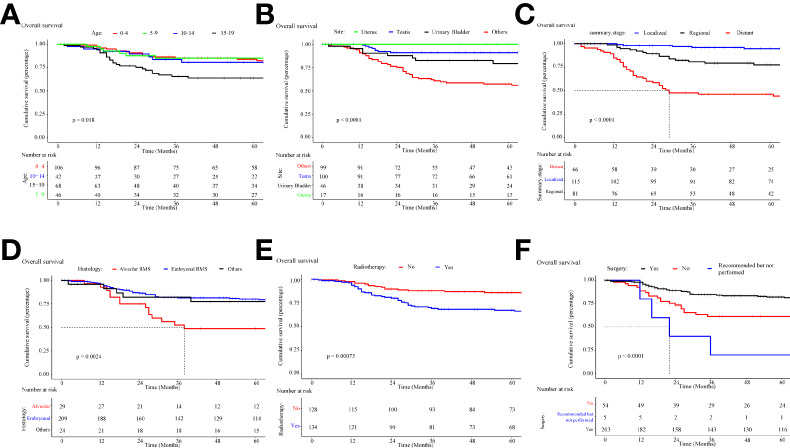
Kaplan-Meier curve of overall survival stratified by **(A)** age, **(B)** site, **(C)** stage, **(D)** histology, **(E)** radiotherapy, **(F)** cancer-directed surgery.

**Figure 8:**
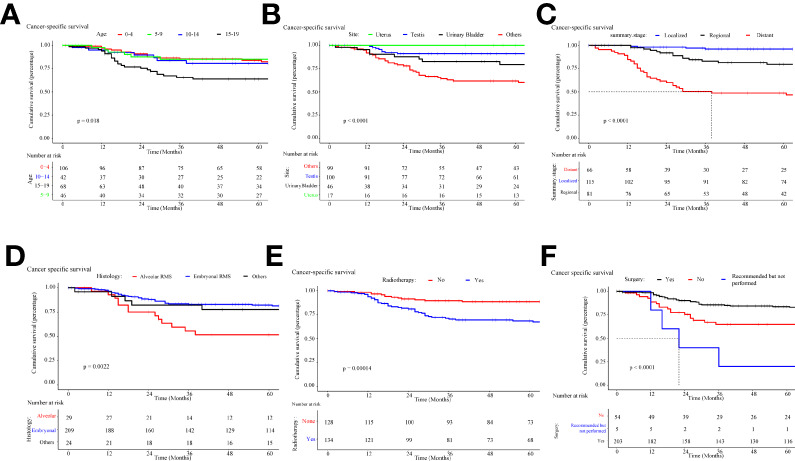
Kaplan-Meier curve of cancer-specific survival stratified by **(A)** age, **(B)** site, **(C)** stage, **(D)** histology, **(E)** radiotherapy, **(F)** cancer-directed surgery.

The overall survival and cancer-specific survival curves are illustrated in **Figures 7**, **8**, respectively. There was a significant difference between the different age cohorts, and the patients in the 15-19 age group had the poorest OS (*P* < 0.05) (**Figure 7A**), and CSS (*P* < 0.05) (**Figure 8A**), which is consistent with the results of the multivariate Cox regression analysis (**Table 2**). Comparing the OS and CSS of patients with different organs involved RMS (**Figures 7B**, **8B**), the uterus, urinary bladder, and testis had better prognostic survival than prostate-involved RMS (*P* < 0.001). In terms of different stages (**Figures 7C**, **8C**), localized-stage RMS showed the best OS and CSS, while distant-stage RMS had the poorest prognostic survival (median OS, 28 months; median CSS, 29 months). There was a significant prognostic difference between the alveolar group and other sub-histological groups (embryonal, etc.) (*P* < 0.005) (**Figures 7D**, **8D**). In the univariate survival analysis of the RT *vs*. the no-RT group, the RT group had poorer survival than the no-RT group (**Figures 7E**, **8E**). Patients with alveolar RMS had the poorest OS and CSS among the assessed histology groups (median OS, 36.2 months). Interestingly, patients who underwent surgery had significantly better prognostic survival (*P* < 0.001) (**Figure 7F**, **Figure 8F**). There was no significant difference in prognostic survival between the different races (*P*>0.05) (Appendix Figures 1A, 2A) and sexes (*P*>0.05) (Appendix Figures 1. B and 2. B).

**Figure 9:**
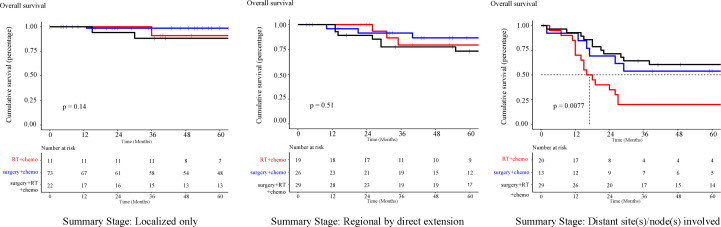
Kaplan-Meier curve of survival stratified by different therapeutic regimens.

On subtype analyses of different therapeutic regimens (**Appendix Tables A1-A3**, **Figure 9**), cancer-directed surgery was associated with improved OS, while treatment with RT in combination with chemotherapy or surgery failed to provide a survival benefit (*P* > 0.05). Interestingly, the significant survival difference between different therapeutic regimens was only observed in patients with distant metastasis, which may be explained by that the majority of patients treated at an early stage of disease can have satisfactory outcomes, while there are higher requirements for therapy options considering the survival of patients with metastasis. The effect of RT on the survival of patients with pelvic and genitourinary RMS requires further investigation.

### Survival outcomes after propensity score analysis

A propensity score analysis was performed to match 55 patients who received RT and 55 who did not receive RT (**Table 3**). As a result, characteristics such as age (*P* =1), site (*P* =0.628), and histology (*P* =0.58) were balanced, without significant differences.

**Table 3:** Characteristics of patients after propensity score matching.

	None (N = 55)	Yes (N = 55)	P.value
**Sex**			
Female	11 (20.0%)	9 (16.4%)	0.805
Male	44 (80.0%)	46 (83.6%)	
**age**			
0-4	27 (49.1%)	27 (49.1%)	1
5-9	9 (16.4%)	9 (16.4%)	
10-14	7 (12.7%)	8 (14.5%)	
15-19	12 (21.8%)	11 (20.0%)	
**race**			
Black	12 (21.8%)	11 (20.0%)	0.883
Other	3 (5.5%)	2 (3.6%)	
White	40 (72.7%)	42 (76.4%)	
**site**			
Others	9 (16.4%)	12 (21.8%)	0.628
Tesis+Uterus+Urinary Bladder	46 (83.6%)	43 (78.2%)	
**histology**			
Alveolar RMS etc.	9 (16.4%)	6 (10.9%)	0.58
Embryonal RMS	46 (83.6%)	49 (89.1%)	
**summary.stage**			
Distant	16 (29.1%)	16 (29.1%)	0.789
Localized	19 (34.5%)	22 (40.0%)	
Regional	20 (36.4%)	17 (30.9%)	
**chemotherapy**			
No	0 (0%)	0 (0%)	1
Yes	55 (100%)	55 (100%)	
**cancer.directed.surgery**			
No	6 (10.9%)	6 (10.9%)	1
Yes	49 (89.1%)	49 (89.1%)	

**Figure 10:**
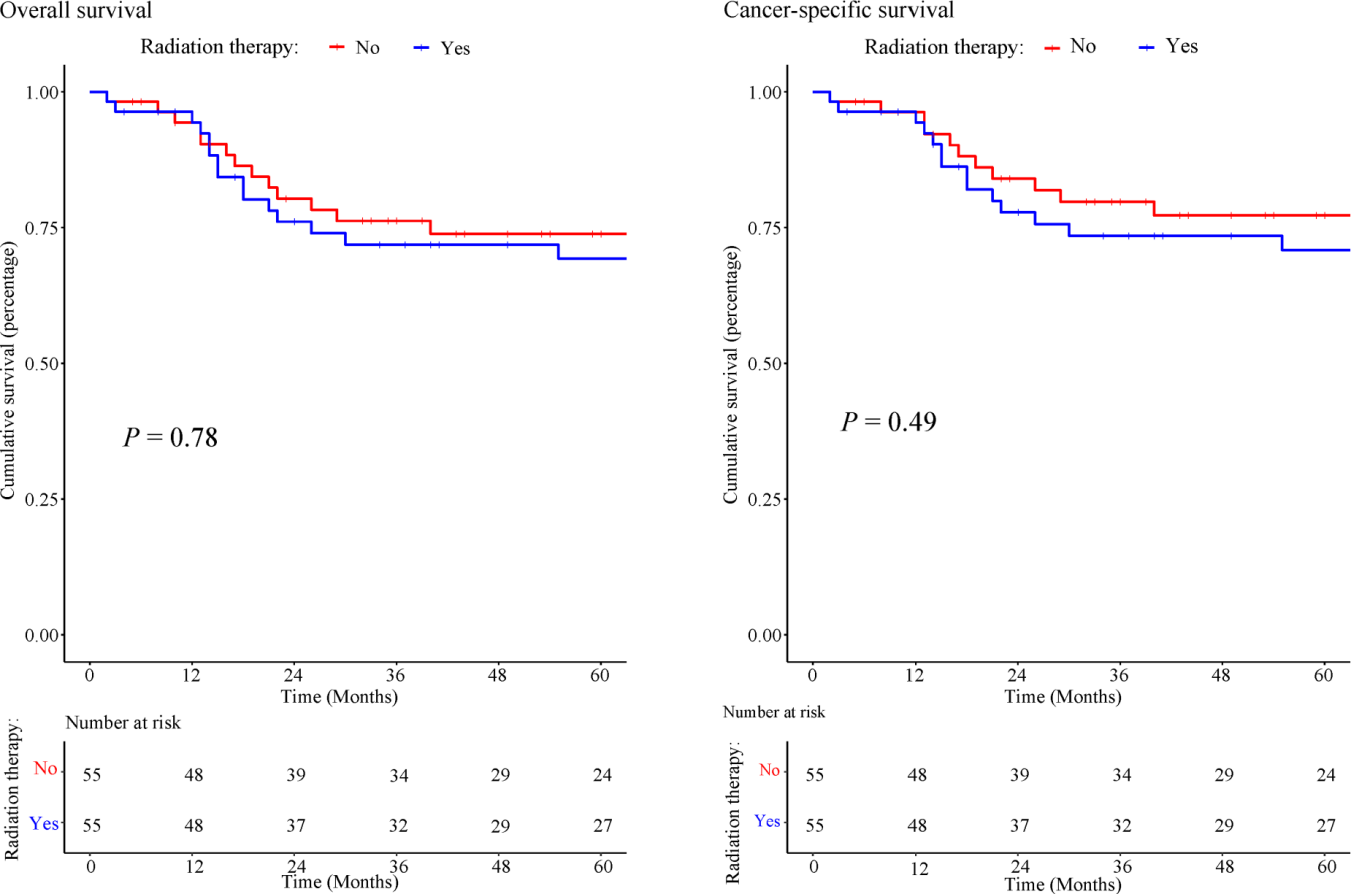
Kaplan-Meier of overall survival and cancer-specific survival in patients treated with or without RT.

Kaplan Meier plot (**Figure 10**) revealed approximately 75% OS at 5 years of follow-up for both the RT and no-RT groups (*P* =0.773) and yielded a statistically insignificant univariable HRs of 1.113 (0.537–2.307, *P* =0.773). The 5-year CSS was approximately 75% for both the RT and no-RT groups (*P* =0.49), with a statistically insignificant univariable HRs of 1.320 (0.599–2.91, *P* =0.49). In multivariable Cox regression analysis (**Table 4**), the implementation of RT was associated with worse prognostic survival, but no statistically significant changes were observed in 5-year survival (OS: HR=1.431; 95% CI: 0.661–3.099; *P* =0.364; CSS: HR=1.601; 95% CI: 0.700–3.662; *P* =0.265).

**Table 4:** Cox regression analysis of the patients after propensity score matching.

Univariate Regression Analysis (OS)		
Risk factor	HR (95% CI)	*P* Value
RT (Yes)	1.113 (0.537- 2.307)	0.773
RT (No)	Ref	
**Univariate Regression Analysis (CSS)**		
RT (Yes)	1.320 (0.599- 2.91)	0.49
RT (No)	Ref	
**Multivariate Cox Proportional Hazards Analysis (OS)**		
RT (Yes)	1.431 (0.661, 3.099)	0.364
RT (No)	Ref	
**Multivariate Cox Proportional Hazards Analysis (CSS)**		
RT (Yes)	1.601 (0.699, 3.662)	0.265
RT (No)	Ref	

Signif. codes: ‘***’ 0.001; ‘**’ 0.01; ‘*’ 0.05, ‘.’ 0.1; ‘ ‘ 1.

## Discussion

RMS lesions in the pelvic cavity and urogenital system are often close to large blood vessels and vital organs and usually grow too large to be completely removed before diagnosis. According to the Children’s Oncology Group (COG) and groups in Europe (the Soft Tissue Sarcoma Committee of the COG, etc.) (12), multimodal therapy combining surgical resection, preoperative/postoperative chemotherapy, and RT has been the overall treatment philosophy. However, there are no guidelines, especially for pediatric pelvic and genitourinary RMS. Therefore, the prognostic factors for pediatric pelvic and genitourinary RMS and the outcomes of different treatments are worth exploring. To the best of our knowledge, this is the first population-based study to determine prognostic factors and assess the outcomes of pediatric patients with pelvic and genitourinary RMS.

This study found that the histological subtype, age, pathological stage, and site were significantly associated with OS and CSS. According to the histological features, RMS can be divided into two main subtypes (embryonal and alveolar) and other rare subtypes (pleomorphic, anaplastic, etc.). In this study, alveolar RMS showed a poorer prognosis than embryonal, pleomorphic, and anaplastic RMS, which is consistent with the recent understanding of pediatric RMS (13–15). It is more difficult to treat alveolar RMS than other subtypes (embryonal type, etc.) due to its discrete location, metastatic tendency and high degree of malignancy. Therefore, in this study, the proportion of patients with alveolar type who received surgery was higher than that of patients with embryonal type. For different age groups, patients in the 15-19 group showed a significantly worse prognostic survival than patients aged 0-15 years (OS: HR 2.984, 95% CI 1.442-6.175, *P* < 0.01**; CSS: HR 2.370, 95% CI 1.257-5.863, *P* < 0.05*). The same report as shown by the Italian and German Soft Tissue Cooperative Groups that age <10 years at diagnosis and embryonal histology are favorable prognostic factors (16, 17). The prognosis of pediatric metastatic RMS remains poor (18). We found that a higher tumor stage was associated with worse prognosis (OS: localized stage: HR 0.070, 95% CI 0.022-0.192, *P* < 0.001; CSS: localized stage: HR 0.065, 95% CI 0.022-0.219, *P* < 0.001). We also found that RMS in the testis and uterus has a much better prognosis than RMS in other locations, suggesting that pediatric RMS in reproductive organs may has better prognostic survival.

For decades, surgery associated with chemotherapy and RT has been the gold standard treatment for patients with RMS (19). Our results support the idea that surgery is the most important therapy in RMS treatment (20), as we found that cancer-directed surgery significantly improved five-year OS/CSS. In our subgroup analysis, chemotherapy improved the survival rate (HR=0.221; 95% CI, 0.056–0.878; *P*=0.032). Although some studies have shown different results in that preoperative and/or postoperative chemotherapy is ineffective (21, 22), chemotherapy continues to be recommended due to surgical benefits (tumor shrinkage after chemotherapy). Collectively, appropriate chemotherapy can confer overall prognostic survival for patients with pelvic and genitourinary RMS.

To the best of our knowledge, this analysis included the largest cohort of children with pelvic and genitourinary RMS treated with RT. The propensity score analysis showed that postoperative RT provided no significant survival benefit for children with pelvic and genitourinary RMS. Similar results were obtained in a study that included 237 patients with vaginal/uterine RMS, and the pooled analysis showed no statistical difference (*P*>0.05) in OS between patients with and without RT (10-year OS: 94% without RT *vs*. 89% with RT) (23). In subgroup Cox analysis, our study also showed that RT failed to provide survival benefits, even with chemotherapy or surgery. Although the clinical efficacy of RT remains to be evaluated, American clinical guidelines for RMS in children still recommend RT as a standard treatment. Approximately 75% of children with RMS are treated with RT, and long-term side effects have frequently been observed at different sites (4). When pelvic radiation is used for pediatric RMS, RT-related toxicity can affect normal tissues, which may result in growth asymmetries, cystitis, infertility, and sexual dysfunction (24–26). Late radiation-induced toxicity also includes decreased bone growth, increased risk of secondary malignancy, and hematuria (27–29). Therefore, we must be aware of the potential toxicity to patients’ lives (25). Currently, at least three randomized clinical trials of pediatric RMS to evaluate the survival impact of RT are in progress (NCT00002995, NCT01626170, and NCT00075582). As data from NCT00002995 have shown, no evidence suggests that reduced RT dose has a negative impact on 5-year failure-free survival (FFS) and OS (localized, stage1/2/3 embryonal RMS, treated with surgical resection and chemotherapy (VA/VAC)) (30). For patients with localized RMS of the vagina, RT-related long-term effects are sometimes unacceptable, especially in children under 24 months of age (31).

Current studies concentrating on the prognostic factors of RMS were mostly based on pathological factors at the time of initial diagnosis, which did not calculate the dynamic changes that occur during the disease process (32). Our study is the first to predict the prognostic survival of pelvic and genitourinary RMS throughout the disease course. We defined different risk factors and constructed relevant nomograms to predict the OS/CSS in patients with pelvic and genitourinary RMS. These nomograms may help predict prognosis more accurately.

Our study has several limitations. Since pediatric RMS is a rare type of pediatric cancer, and the incidence of different subtypes varies greatly (33, 34), there are certain different subtype proportions in this study. The conclusions about alveolar and other rare subtypes need to be validated in more cases in future. Given the retrospective nature of this study, all analyses were subject to selection biases and imbalances in unquantified variables. Of particular importance, specific regimens and dosages for chemotherapy and RT were unavailable. We used several analytical approaches to address potential unmeasured confounding factors, including LASSO regression, multivariable adjustment, and propensity score analysis. All the analytical approaches provided generally consistent results.

In conclusion, age at diagnosis of < 15 years, non-alveolar histological subtype, early tumor stage (localized/regional), specific sites (uterus and testis), and treatment (cancer-directed surgery and chemotherapy) were favorable prognostic factors. The survival nomogram is a user-friendly tool composed of readily available baseline objective data elements that allow robust estimates of survival in patients, overcoming the epistemic uncertainty of the prognostication of this disease. The results of this analysis suggest that RT may not associated with improved prognostic survival in patients with pelvic and genitourinary RMS. Randomized trials to evaluate the impact of RT in pediatric pelvic and genitourinary RMS are warranted. In contrast, cancer-directed surgery can significantly extend life expectancy and increase the cure rate, and chemotherapy may play a role as an adjuvant therapy to improve the curative effects.

## Data availability statement

The original contributions presented in the study are included in the article/**Supplementary Material**. Further inquiries can be directed to the corresponding authors.

## Ethics statement

Ethical review and approval was not required for the study on human participants in accordance with the local legislation and institutional requirements. Written informed consent from the participants’ legal guardian/next of kin was not required to participate in this study in accordance with the national legislation and the institutional requirements.

## Author contributions

JWu and XS designed the research and wrote the paper, JC and JM provided professional advice, and JWu performed the research and analyzed the data. All authors read and commented on the paper. All authors contributed to the article and approved the submitted version.

## Funding

This research was funded by Open Foundation of Key Laboratory of Digital Technology in Medical Diagnostics of Zhejiang Province (Grant No. SZZD202217), National Natural Science Foundation of China (Grant No. 81573516).

## Acknowledgments

We thank Dr. Robert M. Dorazio for providing support in research design and analytical process.

## Conflict of interest

The authors declare that the research was conducted in the absence of any commercial or financial relationships that could be construed as a potential conflict of interest.

## Publisher’s note

All claims expressed in this article are solely those of the authors and do not necessarily represent those of their affiliated organizations, or those of the publisher, the editors and the reviewers. Any product that may be evaluated in this article, or claim that may be made by its manufacturer, is not guaranteed or endorsed by the publisher.

## Abbreviations

RMS, Rhabdomyosarcoma; RT, radiotherapy; SEER, The Surveillance, Epidemiology, and End Results Program; LASSO, least absolute shrinkage and selection operator; OS, overall survival; CSS, cancer-specific survival; C-index, concordance index; AUC, receiver operating characteristic curve; PSM, propensity score matching.

## Appendixes

**Appendix Table A1:** Survival analyses of different therapeutic regimens-localized.

Regimen	Overall Survival			
	HR	95% CI		*P*
Surgery+chemo	0.177	0.011 to 2.823		0.220
Surgery+ RT+chemo	1.357	0.123 to 14.970		0.803
RT+chemo	Reference		Reference	

**Appendix Table A2:** Survival analyses of different therapeutic regimens-regional.

Regimen	Overall Survival			
	HR	95% CI		*P*
Surgery+chemo	0.706	0.143 to 3.503		0.671
Surgery+ RT+chemo	1.514	0.391 to 5.862		0.548
RT+chemo	Reference		Reference	

**Appendix Table A3:** Survival analyses of different therapeutic regimens-distant.

Regimen	Overall Survival			
	HR	95% CI		*P*
Surgery+chemo	0.444	0.182 to 1.087		0.077.
Surgery+ RT+chemo	0.323	0.151 to 0.690		0.004 **
RT+chemo	Reference		Reference	
