# Supplementary figures and images for: Prognostic factors of pediatric pelvic and genitourinary rhabdomyosarcoma: An analysis based on SEER database
Source: Front Oncol. 2022 Sep 5;12:992738. doi: 10.3389/fonc.2022.992738 (PMC9483154; doi:10.3389/fonc.2022.992738)

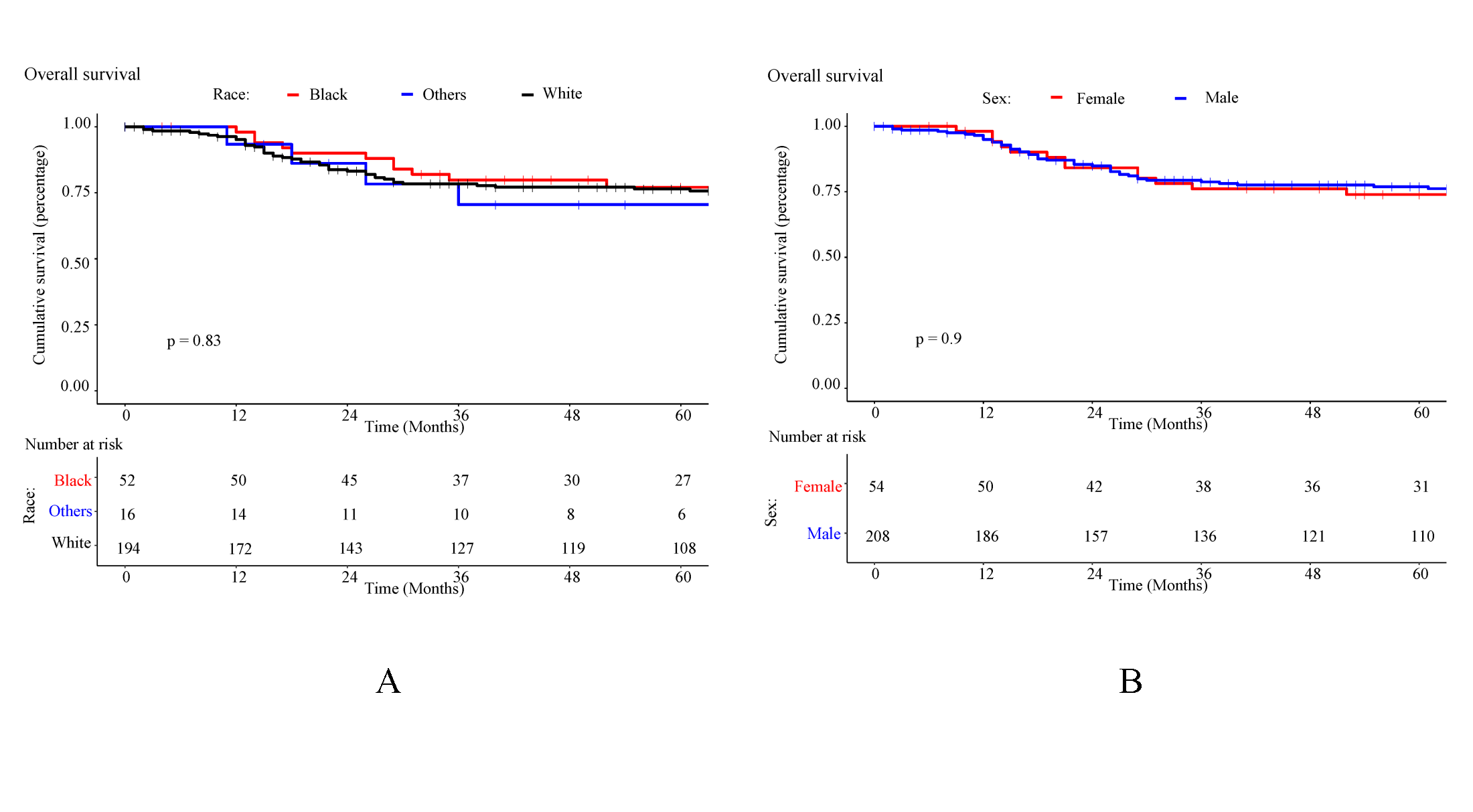

Supplement: Supplementary file 2 [file Image_1.tiff]

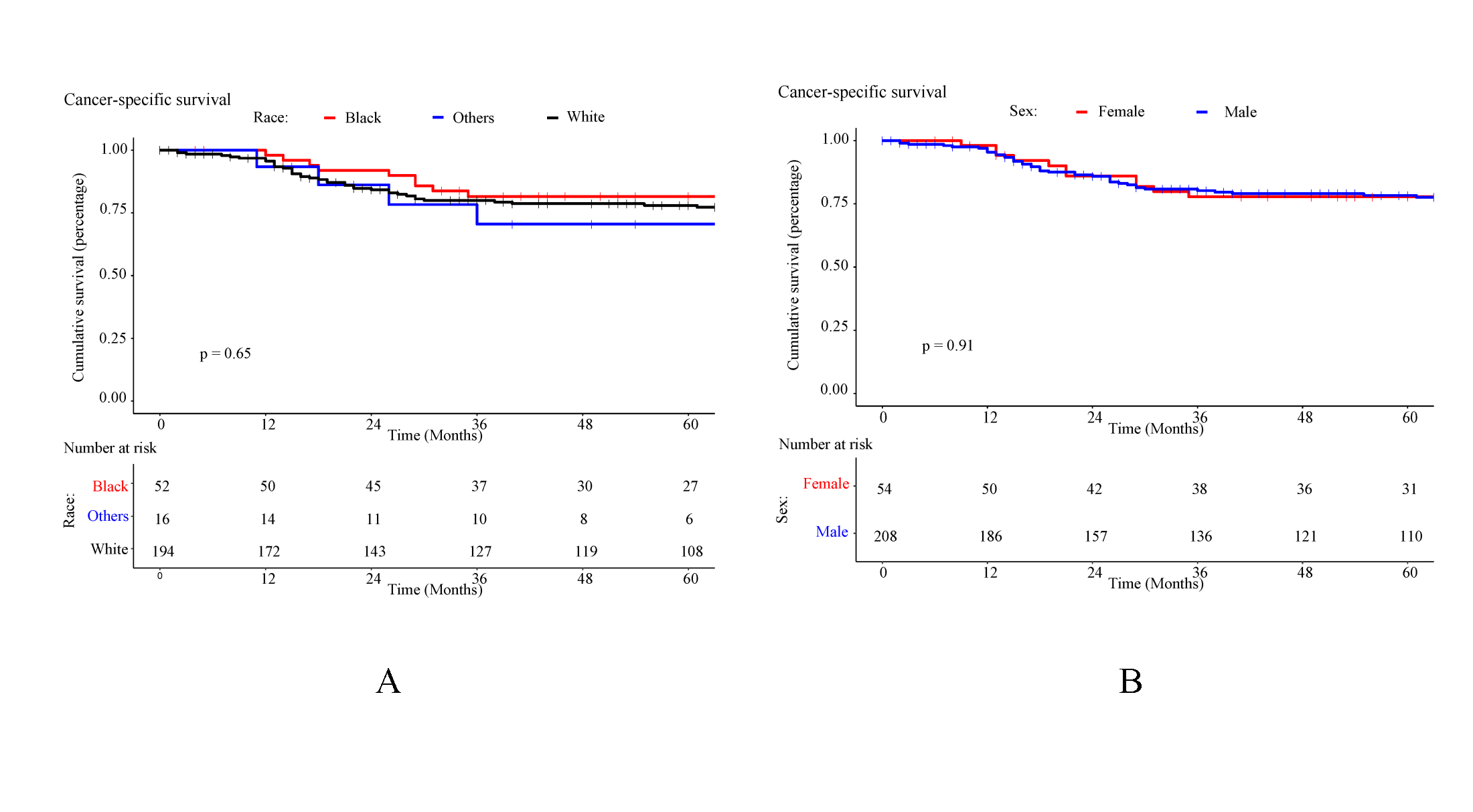

Supplement: Supplementary file 3 [file Image_2.tiff]
